# Reduced RG-II pectin dimerization disrupts differential growth by attenuating hormonal regulation

**DOI:** 10.1126/sciadv.ads0760

**Published:** 2025-02-12

**Authors:** Pawan Kumar Jewaria, Bibek Aryal, Rifat Ara Begum, Yaowei Wang, Gloria Sancho-Andrés, Abu Imran Baba, Meng Yu, Xiaojuan Li, Jinxing Lin, Stephen C. Fry, Stephane Verger, Eugenia Russinova, Kristoffer Jonsson, Rishikesh P. Bhalerao

**Affiliations:** ^1^ Department of Forest Genetics and Plant Physiology, SLU, S-901 83 Umeå, Sweden.; ^2^ Beijing Advanced Innovation Center for Tree Breeding by Molecular Design, Beijing Forestry University, Beijing 10083, China.; ^3^ National Institute of Plant Genome Research, Aruna Asaf Ali Marg, New Delhi 110067, India.; ^4^ Department of Biochemistry and Molecular Biology, Faculty of Biological Sciences, University of Dhaka, Curzon Hall, Dhaka 1000, Bangladesh.; ^5^ The Edinburgh Cell Wall Group, Institute of Molecular Plant Sciences, The University of Edinburgh, Daniel Rutherford Building, The King’s Buildings, Edinburgh EH9 3BF, UK.; ^6^ Department of Plant Biotechnology and Bioinformatics, Ghent University, 9052 Ghent, Belgium.; ^7^ Center for Plant Systems Biology, VIB, 9052 Ghent, Belgium.; ^8^ Department of Biology, ETH Zurich, Zurich 8092, Switzerland.; ^9^ College of Life Sciences, Hebei Agriculture University, 071001 Baoding, China.; ^10^ Umeå Plant Science Centre (UPSC), Department of Plant Physiology, Umeå University, Umeå 90187, Sweden.; ^11^ RBV, Department of Biological Sciences, University of Montreal, 4101 Sherbrooke East, Montreal, QC H1X 2B2, Canada.

## Abstract

Defects in cell wall integrity (CWI) profoundly affect plant growth, although, underlying mechanisms are not well understood. We show that in *Arabidopsis mur1* mutant, CWI defects from compromising dimerization of RG-II pectin, a key component of cell wall, attenuate the expression of auxin response factors *ARF7-ARF19*. As a result, polar auxin transport components are misexpressed, disrupting auxin response asymmetry, leading to defective apical hook development. Accordingly, *mur1* hook defects are suppressed by enhancing *ARF7* expression. In addition, expression of brassinosteroid biosynthesis genes is down-regulated in *mur1* mutant, and supplementing brassinosteroid or enhancing brassinosteroid signaling suppresses *mur1* hook defects. Intriguingly, brassinosteroid enhances RG-II dimerization, showing hormonal feedback to the cell wall. Our results thus reveal a previously unrecognized link between cell wall defects from reduced RG-II dimerization and growth regulation mediated via modulation of auxin-brassinosteroid pathways in early seedling development.

## Teaser

Cell wall integrity defects disrupt apical hook development by acting on auxin-brassinosteroid pathways.

## INTRODUCTION

Plant cells are enclosed by a cell wall, a complex structure generated by intricate interactions between cell wall polymers (*1*). The cell wall strengthens the cell’s structural integrity and functions as a barrier against pathogens. There is growing evidence that the cell wall functions as a hub for mechanochemical signaling and plays a crucial role in morphogenetic responses to developmental cues (*2*, *3*). Also, several components mediating the control of morphogenesis in response to signaling from the cell wall have been identified in plants (*4*–*8*).

The main components of the primary cell wall are cellulose, hemicelluloses such as xyloglucan, and pectin. Interactions between these components strongly affect the wall’s mechanochemical properties (*9*). Unlike cellulose and xyloglucans, pectin is a highly complex polymer with three subdomains: homogalacturonan (HG), rhamnogalacturonan-I (RG-I), and RG-II. RG-II is highly branched, with multiple side chain modifications (*10*, *11*). RG-II dimerizes via the formation of cross-linking boron bridges between apiose residues in its side chains (*12*–*14*). RG-II dimerization has a profound impact on plant growth as revealed by mutants of the *MUR1* gene, which is required for the generation of guanosine 5′-diphosphate (GDP)–fucose (and thus for incorporating fucose into RG-II) (*13*). The *mur1* mutant has normal levels of RG-II, but the two fucosyl residues in its side chain are replaced by galactosyl residues and consequently, in the *mur1* mutant, both the rate of RG-II dimerization formation and stability are highly compromised, with the majority of RG-II existing as a monomer. Consequently, the *mur1* mutant displays severe growth defects that can be completely suppressed by treatment with exogenous boron, which restores RG-II dimerization in the *mur1* mutant (*15*). These results thus clearly highlight the link between RG-II dimerization defects and the growth phenotype of *mur1* mutant and demonstrate the crucial role of RG-II dimerization for plant growth. The reduced RG-II dimerization in *mur1* also results in weaker walls (*16*) and diminished freezing tolerance (*17*). However, *mur1* mutant cells exhibit reduced elongation (*15*), which is somewhat counterintuitive because walls are weaker in the *mur1* mutant and should presumably yield more easily to turgor. Thus, how mechanochemical defects in the *mur1* leading to weaker walls cause growth inhibition remains enigmatic.

We recently discovered that the *mur1* mutants deficient in RG-II dimerization exhibit severe defects in apical hook development. This prompted us to use the apical hook as a model to address the role of defects in RG-II dimerization in plant growth regulation. The bending of the hypocotyl to form the apical hook during seed germination is crucial for successful seedling emergence (*18*). The apical hook is a simple experimental model for studying organ-bending tissue because it is mediated primarily via differential cell elongation (*19*). During apical hook formation studied here, epidermal cells on the hook’s outer side elongate faster than those on the inner side. It was recently shown that this differential growth results from interactions between growth regulators and the cell wall: A strong auxin response on the inner side makes the cell walls there comparatively more resistant to extension, while the walls on the outer side remain more extensible (*18*, *20*), resulting in bending of the hypocotyl. Here, by exploiting the cell wall defects of the *mur1* mutant that results in perturbation of apical hook development, we reveal a link between cell wall defects and hormonal pathways that affect the regulation of differential growth during hypocotyl bending in plants.

## RESULTS

### Reduced RG-II dimerization in *mur1* mutant disrupts apical hook development

**Fig. 1.**
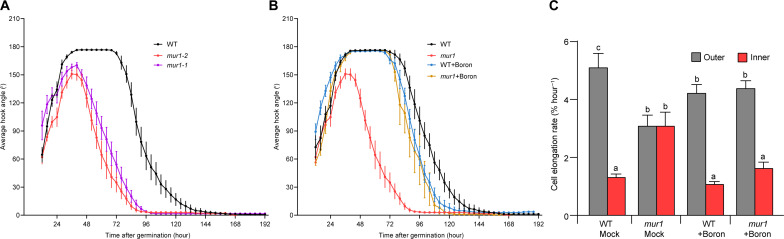
RG-II dimerization plays a key role in apical hook development. (**A**) Kinematics analyses of apical hook development in dark-grown wild-type (WT), *mur1-1*, and *mur1-2* seedlings. For each genotype and condition, *n* ≥ 15. Error bars represent the SE of the mean. (**B**) Kinematics analyses of apical hook development in dark-grown WT and *mur1-2* seedlings without or after supplementation with 250 μM boric acid (boron). Error bars represent the SE of the mean (*n* ≥ 15). (**C**) Cell elongation rates of the outer and inner epidermis of dark-grown WT and *mur1-2* mutant plants without or with supplementation with 250 μM boric acid (boron). The lengths of epidermal cells in the region of 0 to 400 μM from the shoot apical meristem were measured every 2 hours and the plot shows the average cell elongation rate over 8 hours. For each genotype, *n* = 6. Error bars represent the SE of the mean. Significant differences according to Tukey’s post hoc test and Duncan’s test [*P* < 0.05, one-way analysis of variance (ANOVA)] are indicated by different lowercase letters above the columns.

The *mur1* mutant is defective in RG-II dimerization (*15*). To better understand the role of RG-II in morphogenesis, we investigated apical hook development in the *mur1* mutant. Whereas the wild-type forms an apical hook with an angle of 180° that is maintained for almost 72 hours, two independent alleles of the *mur1* mutant (*mur1-1* and *mur1-2*) show severe defects in hook formation: The maximum hook angle in *mur1-1* and *mur1-2* is only 150°, and the hook opens substantially faster than in the wild type (Fig. 1A). As both *mur1* mutant alleles displayed the same phenotype, we subsequently used *mur1-2* mutant allele (henceforth referred to as *mur1*) for further analysis. The *mur1* mutant is deficient in GDP-d-mannose-4,6-dehydratase, which catalyzes the first step in the de novo synthesis of GDP-l-fucose (*13*). Therefore, we first checked whether the *mur1* hook phenotype can be suppressed by supplementing with fucose. Our results indeed show that exogenously added fucose suppresses the *mur1* hook defects (fig. S1A). However, GDP-fucose is not only required for the incorporation of fucose into side branch A of RG-II but also for fucosylation of xyloglucans, catalyzed by MUR2 protein (*21*). Thus potentially, reduced fucosylation in xyloglucan could also contribute to hook defects due to loss of MUR1 (*22*, *15*). To address this possibility, we analyzed hook development in the *mur2* mutant defective in the fucosylation step of xyloglucan biosynthesis. Unlike *mur1*, the *mur2* mutant displayed no hook development defects and was indistinguishable from the wild type (fig. S1B). Thus, the hook defects of *mur1* are presumably due to defects in RG-II dimerization rather than reduced fucosylation of xyloglucan, which is in agreement with data showing RG II dimerization defects rather than reduced fucosylation of xyloglucan is important for the *mur1* phenotype (*16*).

RG-II dimerizes via borate diester bonds (boron bridges) that influence the cell wall architecture. Previous data have shown that RG-II dimerization is highly reduced in *mur1* mutant, and suppression of the mechanical and growth defects in the *mur1* mutant by exogenous boron (supplemented as boric acid) that restores RG-II dimerization indicates that reduced RG-II dimerization contributes to *mur1* growth defects (*15*). We therefore investigated whether restoring RG-II dimerization (by adding boron) can also rescue *mur1* hook defects. Treating *mur1* with exogenous boron rescued the *mur1* mutant’s hook defects and made its phenotype indistinguishable from the wild type (Fig. 1B). Conversely, treatment with the boric acid derivative phenylboronic acid (PBA) to mimic boron depletion conditions (*23*, *24*) strongly perturbed hook development in the wild type, phenocopying the *mur1* mutant’s hook defects (fig. S2). The genetic and pharmacological evidence showing suppression of the *mur1* hook defects by restoring RG-II dimerization strongly links reduced RG-II dimerization with hook defects in the *mur1* mutant.

### Growth asymmetry crucial for apical hook development is disrupted in *mur1* mutant

The apical hook forms as a result of differential growth on opposite sides of the hypocotyl: Growth is rapid on the outer side and restricted on the inner side. To determine the underlying cause of the hook defects in the *mur1* mutant, we compared cell elongation on the outer and inner sides of the hook in the *mur1* mutant and the wild type. Cell elongation was significantly slower in the *mur1* mutant than in the wild type on the outer side of the hook, and the opposite was true on the inner side (Fig. 1C). The hook defects of *mur1* mutant thus result from disruption of differential growth between the inner and outer sides.

Because boron-mediated restoration of RG-II dimerization rescued hook defects in *mur1*, we investigated whether boron treatment also restores differential growth in *mur1* mutant. Boron treatment restored higher growth on the outer side and growth repression on the inner side of the hook in *mur1* mutant, thereby re-establishing the wild type differential growth pattern (Fig. 1C). Thus, restoring RG-II dimerization defects (by boron addition) suppresses differential growth defects in the *mur1* mutant.

### The auxin response maximum is attenuated in the *mur1* mutant

**Fig. 2.**
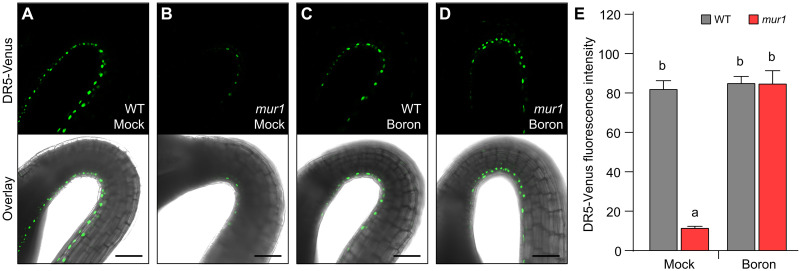
Reduced RG-II dimerization perturbs auxin response maxima. (**A** to **D**) Auxin response maxima on the inner and outer sides of the apical hook were visualized using the synthetic auxin response reporter DR5-Venus in the WT and the *mur1-2* mutant without or with supplementation with 250 μM boric acid (boron). Scale bars, 20 μm. (**E**) The fluorescence intensity of the Venus protein in the apical hook with and without boron in the WT and *mur1-2* was then analyzed. The plotted values are means ± SE (*n* ≥ 60 to 70 cells from 10 seedlings for each genotype). Significant differences according to Tukey’s post hoc test and Duncan’s test (*P* < 0.05) are indicated by different lowercase letters.

An auxin response maximum on the inner side of the hook is crucial for repressing growth and thus for proper apical hook development (*25*). Therefore, we investigated whether the loss of growth repression on the inner side in the *mur1* mutant is due to attenuation of this auxin response maximum. In accordance with previous reports, visualizations of the activity of the synthetic auxin response reporter DR5-Venus in the wild type revealed a significantly more intense auxin response maximum on the inner side of the hypocotyl compared to the outer side (Fig. 2A). However, the DR5-Venus signal intensity in cells on the inner side in the *mur1* mutant is significantly reduced compared to the wild type, indicating severe attenuation of the auxin response maximum (Fig. 2, B and E). Treatment with boron restored the inner side auxin response maximum in the *mur1* mutant to the wild type level (Fig. 2, D and E). Thus, auxin response maxima crucial for hook development are attenuated in *mur1* mutant, which may explain its hook defects.

### Polar auxin transport in apical hook is disrupted in *mur1* mutant

**Fig. 3.**
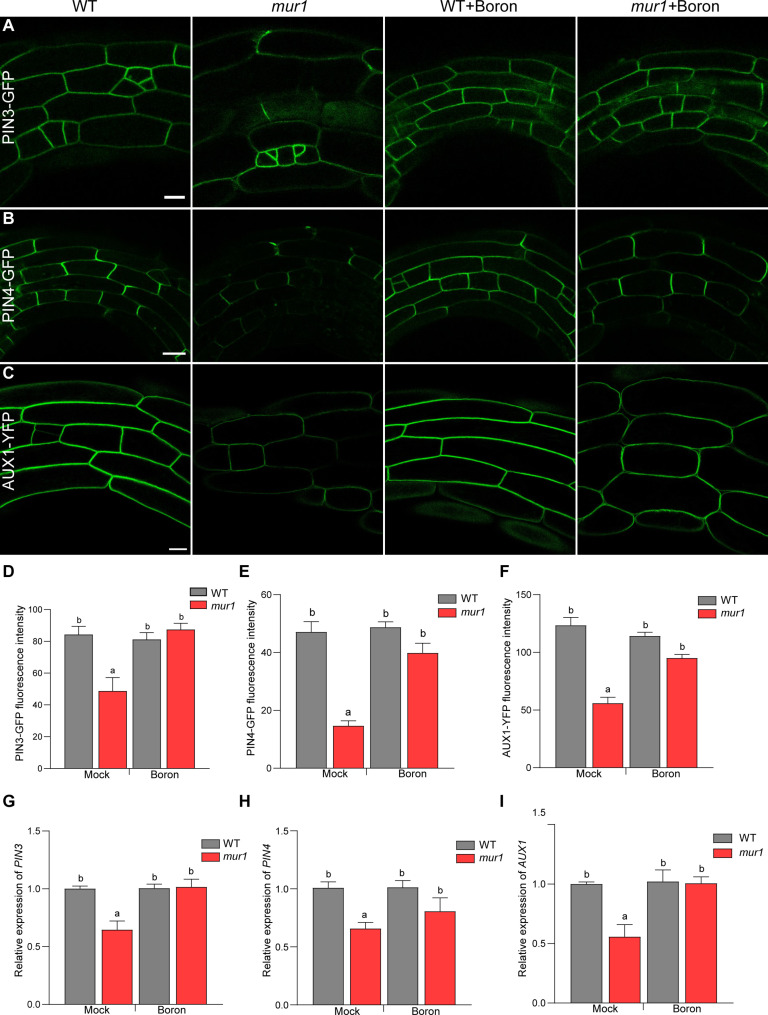
Plasma membrane levels of auxin transport carriers are altered in *mur1-2* mutant. Representative confocal images of the plasma membrane of (**A**) PIN3-GFP, (**B**) PIN4-GFP, and (**C**) AUX1-YFP in the apical hook of WT and *mur1-2* mutant in the presence and absence of boric acid (boron). Scale bars, 20 μm. Quantification of plasma membrane fluorescence intensity of (**D**) PIN3-GFP, (**E**) PIN4-GFP, and (**F**) AUX1-YFP in WT and *mur1-2* mutant in the presence and absence of 250 μM boric acid. Seedlings were imaged 48 hours after germination. For each genotype and treatment, five cells from each of the 10 seedlings were analyzed. Significant differences according to Tukey’s post hoc test and Duncan’s test (*P* < 0.05) are indicated by different lowercase letters. (G to I) Transcript levels of (**G**) *PIN3*, (**H**) *PIN4*, and (**I**) *AUX1* in WT, *mur1-2* mutant in the presence and absence of 250 μM boric acid (boron). Graphs represent averages of three biological replicates. Ubiquitin was used as an internal control. Plotted values are averages for three independent biological replicates. Significant differences according to Tukey’s post hoc test and Duncan’s test (*P* < 0.05) are indicated by different lowercase letters.

The key components of polar auxin transport machinery, PIN and AUX/LAX genes, are essential for auxin response maxima in the apical hook. Mutants in *PIN* and *AUX/LAX* genes have disruption of auxin response maxima and thus exhibit notable hook defects (*25*, *26*). The disruption of auxin response maxima in *mur1* mutant prompted us to investigate whether the reduction of RG-II dimerization affects polar auxin transport machinery. Therefore, we compared the plasma membrane levels of PIN and AUX/LAX proteins in *mur1* mutant with that of wild type in the hook. The plasma membrane levels of PIN3, PIN4, and AUX1 were significantly reduced in the *mur1* mutant (Fig. 3, A to F), whereas that of LAX3 was not (fig. S3). Supplementing the *mur1* mutant with boron restored the PIN3, PIN4, and AUX1 levels to wild type (Fig. 3, A to F). Gene expression analysis indicated that the reduction of PIN3, PIN4, and AUX1 levels in *mur1* mutant was due to a reduction in their transcript levels (Fig. 3, G to I). Moreover, the reduction of *PIN3*, *PIN4*, and *AUX1* transcripts in *mur1* mutant could be suppressed by the addition of exogenously added boron which restores RG-II dimerization in *mur1* mutant. These results suggest that reduced RG-II dimerization contributes to the disruption of polar auxin transport, resulting in attenuation of auxin response maxima in *mur1* mutant.

### Attenuated expression of *ARF7* and *ARF19* contributes to *mur1* hook defects

**Fig. 4.**
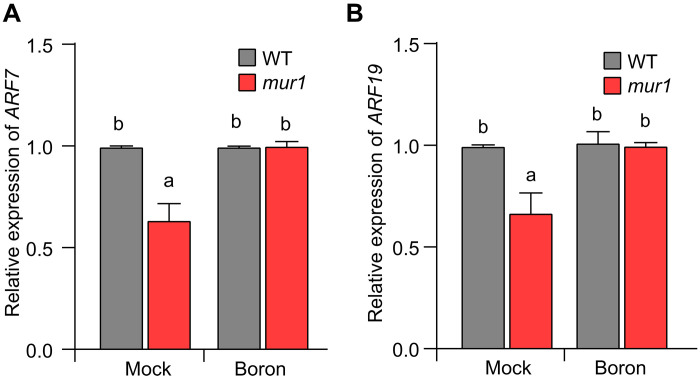
The *mur1* hook defect is mediated by the ARF7 and ARF19 pathway. (**A** and **B**) qRT-PCR analysis of *ARF7* (A) and *ARF19* (B) expression in the WT and the *mur1* mutant in the presence and absence of 250 μM boric acid (boron). Expression of *ARF7* and *ARF19* relative to ubiquitin is plotted on the *y* axis. Plotted values are averages for three independent biological replicates. Significant differences according to Tukey’s post hoc test and Duncan’s test (*P* < 0.05) are indicated by different lowercase letters.

The redundantly acting auxin response factors ARF7 and ARF19 play a major role in hook development (*25*) and the *arf7/arf19* double mutant exhibits a perturbed auxin response and severe hook defects like those of the *mur1* mutant. We therefore investigated the expression of *ARF7* and *ARF19* in *mur1* mutant and observed significantly reduced expression of both the genes (Fig. 4, A and B). Moreover, treatment of *mur1* mutant with boron restored their expression to wild type levels (Fig. 4, A and B). These results show that reduction in RG-II dimerization results in attenuated expression of *ARF7/ARF19*, crucial regulators of hook development.

In *mur1* mutant, auxin response asymmetry is disrupted due to misexpression of *PIN3*, *PIN4*, and *AUX1*. Therefore, we investigated whether attenuation of *PIN3*, *PIN4*, and *AUX1* expression was linked with reduced *ARF7* and *ARF19* expression. The analysis of the *arf7/arf19* mutant showed that transcript levels of *PIN3*, *PIN4*, and *AUX1* were significantly reduced in the *arf7/arf19* mutant (fig. S4). Together, these results strongly suggest that attenuation of ARF7- and ARF19-dependent auxin pathway contributes to hook defects resulting from the reduction of RG-II dimerization in *mur1* mutant.

To test whether attenuated *ARF* expression contributes to defects in hook development, we expressed *ARF7-mCherry* under epidermis-specific ML1 promoter in *mur1* mutant (thereby enhancing total *ARF7* expression) and analyzed hook development (fig. S5). Driving the *ARF7-mCherry* expression in the epidermis could completely suppress the hook defects of the *mur1* mutant. Together, these results show that ARF7 and ARF19, key regulators of hook development, are the downstream targets of the pathway that links mechanochemical perturbation in cell wall and defective hook development in *mur1* mutant.

### Expression of BR biosynthetic genes is down-regulated in the *mur1* mutant

**Fig. 5.**
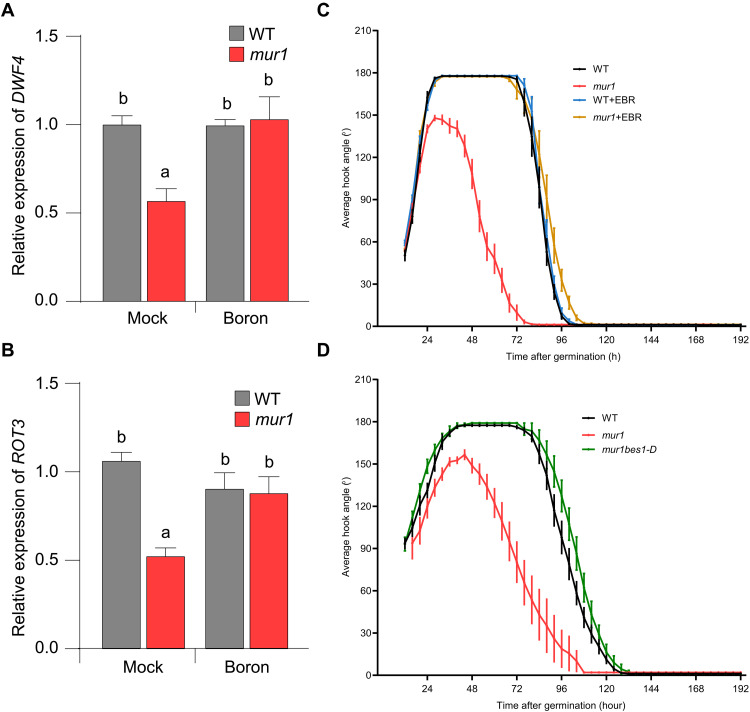
Hook defects resulting from reduced RG-II dimerization are associated with attenuation of the BRs pathway. (**A**) The transcript level of the BR biosynthetic genes *DWF4* and (**B**) *ROT3* expression in the WT and *mur1-2* mutant in the presence or absence of exogenous boron (250 μM boric acid). The values on the *y* axis indicate expression relative to ubiquitin and are averages of three independent biological replicates. Significant differences according to Tukey’s post hoc test and Duncan’s test (*P* < 0.05) are indicated by different lowercase letters. (**C**) Kinematics analyses of apical hook development in dark-grown WT and *mur1-2* seedlings with and without 24-epibrassinolide (EBR) (100 nM). (**D**) Kinematics analyses of apical hook development in dark-grown WT, *mur1-2*, and *bes1-D/mur1-2* seedlings (the gain-of-function mutant *bes1-D* suppress *mur1* hook defect). For each genotype and condition, *n* ≥ 15. Error bars represent the SE of the mean.

Brassinosteroids (BRs) like auxin are also involved in hook development as shown by hook defects similar to those seen in the *mur1* mutant, caused by blocking BR biosynthesis with inhibitors such as brassinazole (BRZ) (*27*). Moreover, there is significant cross-talk between the BR and auxin signaling pathways during hook development (*28*–*30*). Because BRs have also been implicated in cell wall–mediated signaling (*31*), we investigated their role in the hook defects of *mur1* mutant. By measuring the expression of BR biosynthetic genes, we discovered that transcript levels of *DWARF 4* (*DWF4*) (*32*) and *ROTUNDIFOLIA3* (*ROT3*) (*33*) were significantly reduced in *mur1* mutant (Fig. 5, A and B). Moreover, the down-regulation of these genes in the *mur1* mutant could be reversed by treatment with exogenous boron (Fig. 5, A and B). These results suggest that transcriptional down-regulation of genes encoding enzymes in BR biosynthesis contributes to the *mur1* hook defects.

We then tested whether hook defects in the *mur1* mutant are due to the down-regulation of BR biosynthesis–related genes by supplementing the *mur1* mutant with BRs. The *mur1* hook defect was largely suppressed by exogenous 24-epibrassinolide (EBR) (Fig. 5C). We further confirmed the role of BRs in RG-II–mediated hook development by introducing the gain of function *bes1-D* mutant allele in *mur1* background. BES1 accumulates in the nucleus in response to BRs to promote downstream responses. The *bes1-D* is a semidominant mutant allele in which BR response is constitutively active independently of BR (*34*). The introduction of *bes1-D* into the *mur1* mutant completely suppressed the *mur1* hook defects (Fig. 5D), further indicating that attenuation of the BR pathway contributes to hook defects in the *mur1* mutant. Conversely, boron-mediated hook defect suppression of the *mur1* mutant was blocked by the BR biosynthesis inhibitor BRZ (*27*) (fig. S6).

Since cross-talk between BR and auxin has been implicated in hook development (*28*), we investigated whether the transcriptional down-regulation of BR biosynthesis–related genes is due to attenuation of ARF7 and ARF19 activity in the *mur1* mutant. The expression of both *DWF4* and *ROT3* was reduced in the *arf7arf19* double mutant, which phenocopies the *mur1* mutant (fig. S7, A and B). Thus, these results suggest that compromising RG-II dimerization results in the down-regulation of BR biosynthesis–related genes presumably due to the attenuation of *ARF7* and *ARF19* expression and lead to hook defects in the *mur1* mutant.

### Increasing RG-II dimerization enhances BR signaling

Mechanochemical changes in the cell wall are known to modulate BR signaling (*30*). Therefore, we biochemically investigated the role of BR signaling in *mur1*. BES1 is a key component of BR signaling (*34*) and the ratio of phosphorylated BES1 to total BES1 has been used as a sensitive readout for BR signaling activation: A low ratio, i.e., a high relative abundance of the dephosphorylated BES1 protein indicates enhanced BR signaling and vice versa (*34*). We found no significant difference in this ratio between the wild type and the *mur1* mutant, indicating that BR signaling per se was not reduced in the *mur1* mutant. However, the addition of boron (which enhances RG-II dimerization), significantly increased the ratio of dephosphorylated to total BES1 in both the wild type and *mur1* (fig. S8). Moreover, the ratio after boron treatment was lower in the *mur1* mutant background than in the wild type (fig. S8). These results suggest that enhancing the level of RG-II dimerization enhances BR signaling and can thus contribute to the suppression of *mur1* hook defect.

### BRs feed back onto RG-II dimerization in hook development

Hormones affect cell wall chemistry in various ways, for example, auxin loosens cell walls by acidification (*35*, *36*) and also modulates pectin methylesterification levels resulting in modulation of cell elongation (*18*). Because suppression of the *mur1* phenotype requires the restoration of RG-II dimerization, we hypothesized that BRs might contribute to this suppression by altering RG-II dimerization and thereby modulating cell wall properties. Accordingly, we discovered that treatment with exogenous EBR enhanced RG-II dimerization, which may partly explain the suppression of hook defects in *mur1* following EBR treatment (fig. S9). Moreover, EBR-mediated suppression of the *mur1* phenotype was blocked when the boron depletion was mimicked by PBA (fig. S10), further supporting the connection between BR-mediated suppression of *mur1* and boron-mediated RG-II dimerization. These results strongly suggest that enhancing BR signaling can positively feedback onto the cell wall via RG-II dimerization.

### Transduction of cell wall defects in *mur1* is independent of *THESEUS1* and *RLP44*

Perturbation of cell wall mechanical properties affects growth mediated by cell wall integrity (CWI) sensing. Receptor-like kinase THESEUS1 (THE1) has been implicated in mediating CWI sensing (*37*, *38*) and loss of THE1 can suppress hook defects resulting from cell wall alterations caused by reduced cellulose biosynthesis or pectin levels (*39*). Similarly, RLP44/CNU2 mediates changes in pectin methylesterification (*40*) and loss of RLP44 can suppress hook defects stemming from altered pectin methylesterification (*39*). Therefore, we investigated whether either of these two key mediators of CWI defects linked with hook development also mediates in hook defects stemming from reduced RGII pectin dimerization in the *mur1* mutant. To test this, we introduced loss of function *the1-1* and *rlp44* into the *mur1* mutant and then investigated hook development in the *mur1/the1-1* and *mur1/rlp44* double mutants. Both, *mur1/the1-1* and *mur1/rlp44* were indistinguishable from *mur1* mutant (fig. S11). These results suggest that the cell wall defects resulting in disruption of hook development in *mur1* mutant are transduced independently of the pathway involving THE1 or RLP44.

## DISCUSSION

Mechanochemical changes in cell wall properties play a key role in plant morphogenesis. Consequently, defects in cell walls profoundly affect plant growth. However, the link between cell wall defects and downstream pathways and how they affect growth are not fully understood. Using *mur1* mutant in which RG-II dimerization is defective, we demonstrate how perturbation of mechanochemical properties of the cell wall is transduced to modulate hormonal pathways crucial for hypocotyl bending.

RG-II and its boron-mediated dimerization have profound effects on growth (*15*). The small stature of the *mur1* mutant in RG-II dimerization indicates that reduced RG-II dimerization leads to cell elongation defects. Accordingly, growth on the outer side of the apical hook in the *mur1* mutant is reduced relative to the wild type. Intriguingly though, there is a simultaneous failure to repress growth on the inner side in *mur1* mutant, and cell elongation is enhanced on the inner side in the *mur1* apical hooks compared to that in the wild type where it is repressed. Together, *mur1* mutation results in disruption of differential growth. In the wild-type apical hook, auxin response asymmetry plays a crucial role in the regulation of differential growth. The auxin response asymmetry crucial for differential growth is regulated by polar auxin transport machinery that channels auxin to the inner side to generate the auxin response maxima. Our data show that plasma membrane levels of PIN3, PIN4, and AUX1, key components of polar auxin transport are attenuated in the *mur1* mutant. Consequently, the auxin response maximum is disrupted in the *mur1* mutant, which then results in defects in differential growth and resultant hook defects. Our discovery that suppression of boron-sensitive RG-II dimerization defects can restore auxin response maxima in *mur1* reveals yet another facet of the interplay between the cell wall and auxin. For example, high auxin levels were previously shown to increase the relative stiffness of the inner side cell walls by increasing pectin methylesterification (*18*), whereas auxin-mediated cell wall loosening has also been described (*35*, *36*, *41*). Here, we show that the cell wall defects can influence the regulation of auxin response maximum via transcriptional control of polar auxin transport mediated by the redundantly acting auxin response factors ARF7 and ARF19. The expression of *ARF7/ARF19* is reduced in the *mur1* mutant and conversely, enhancing *ARF7* expression suppresses hook defects in the *mur1* mutant. Furthermore, suppression of hook defects in the *mur1* mutant by boron-mediated RG-II dimerization depends on functional ARF7 and ARF19. These results suggest that ARF7/ARF19 are downstream targets of signaling pathway transducing mechanochemical change in cell wall resulting from perturbation of RG-II dimerization that leads to hook defects in the *mur1* mutant. Hence, these findings reveal a link between boron-sensitive RG-II dimerization and auxin-mediated differential growth via ARF7/ARF19 pathway.

In addition to auxin, transcriptional down-regulation of BR biosynthesis–related genes *DWF4* and *ROT3* in the *mur1* mutant suggests a link between cell wall defects and the BR pathway. This is further supported by the restoration of *DWF4* and *ROT3* expression to wild type levels in the *mur1* mutant by boron treatment that corrects RG-II dimerization defects in the *mur1* mutant. A link between boron deficiency and reduced BR response and growth defects has been reported in Arabidopsis roots (*42*). Together, our data showing the negative impact of reduced RG-II dimerization on the expression of BR biosynthesis contributing to the *mur1* mutant’s hook defects are further supported by the suppression of the *mur1* hook phenotype both by treatment with BR and by enhancement of BR signaling by constitutively active BES1. Our data show that enhancing RG-II dimerization has a positive effect on BR signaling. Previously, blocking pectin methylesterification has also been shown to enhance BR signaling (*31*). Thus, our data further highlight a close link between the response of the BR pathway and changes in the cell wall composition. In addition, our data show that blocking BR response results in a failure of boron to suppress the *mur1* hook defects and supports BR as a downstream component in hook development responding to cell wall perturbations such as reduced RG-II dimerization. In addition, the enhancement of RG-II dimerization by BR, and data showing that BR-mediated suppression of *mur1* hook defects depends on RG-II dimerization reveal a feedback loop between BR and RG-II dimerization.

Our results link the BR pathway with auxin signaling because BR biosynthetic genes are down-regulated in the *mur1* mutant and are also down-regulated in the *arf7arf19* double mutant. Before this study, ARF7 and ARF19 were shown to regulate hook development via their role as key transcriptional regulators of the auxin response pathway (*25*). However, our data now reveal their additional role in hook development via transcriptional control of BR biosynthetic genes. Interactions between the auxin and BR pathways have been reported in several developmental processes (*43*) including hypocotyl elongation (*44*, *45*), tropic responses (*46*), vascular development (*47*), and lateral root development (*48*), and meristem maintenance (*49*). Also, auxin regulates BR biosynthesis, e.g., by regulation of *DWF4* expression (*50*). Two components of the auxin response pathway, namely, the transcriptional repressor ARF2 (a negative regulator of BR response) and the transcriptional activator ARF7, were identified as key points of intersection between auxin and BR signaling (*29*, *47*). Moreover, BRs have also been linked with polar auxin transport regulation in roots (*45*, *51*, *52*). Our results not only provide an additional example of auxin-BR cross-talk but reveal that the auxin and BR pathway interaction is targeted by cell wall perturbation via ARF7 and ARF19 transcription factors.

Several studies have shown the link between pectin, mechanics, and growth control. Changes in levels of pectin affect cell adhesion causing alteration in the mechanical properties of hypocotyl and affecting its growth (*53*). In addition, increasing or decreasing levels of pectin methylesterification affects wall elasticity and indicates a role for pectin methylesterification in processes such as organ initiation, hypocotyl elongation as well as pavement cell lobing (*54*–*58*). Hormonal control of cell wall mechanochemical properties mediated via pectin methylesterifcation plays a critical role in hook development (*18*). It was shown that auxin-regulated methylesterification of HG pectin (another pectin subcomponent) stiffens cell walls to repress growth on the inner side of the hook (*18*). Here, we reveal the effect of cell wall perturbation due to reduced RG-II dimerization on the regulation of hormonal pathways that control hook development. Earlier studies demonstrated the role of pectin methylesterification in cell wall–mediated control of auxin distribution via its impact on the polarity of auxin transporters (*18*, *59*). Our results reveal how a change in cell wall composition results in disruption of hook development summarized in a model (fig. S12). On the basis of these results, we propose a link between reduced RG-II dimerization and resultant modulation of transcriptional control of polar auxin transport and also of another BR pathway (via down-regulation of *ARF7/ARF19* expression), resulting in disruption of growth asymmetry and defects in hook development.

CWI defects often lead to a bursting of cells (*4*, *60*–*62*). Cell wall defect can also affect growth as in the case of *mur1* mutant, although the underlying mechanisms are not always well understood. The receptor-like kinase THESEUS1 and RLP44 have previously been shown to link CWI defects with disruption of hook development, as their inactivation can suppress hook defects resulting from cell wall alterations. However, our findings indicate that neither *the1* nor *rlp44* mutants can similarly suppress *mur1* hook defects. These results indicate that cell wall defects arising from RG-II dimerization may be recognized through a pathway independent of THE1 or RLP44, reflecting the complexity of the CWI sensing system. In addition to THE1, receptor like-kinases such as FERONIA (FER) and RESISTANCE TO FUSARIUM OXYSPORUM (RFO) have been shown to mediate in transducing cell wall defects stemming from altered pectin composition (*7*, *60*). Whether FER, RFO, or other receptor-like kinases also play a role in sensing the defects resulting from compromised RG-II dimerization remains to be seen. Interactions between RALF peptides, LORELEI-LIKE-GPI-ANCHORED PROTEIN 1 (LLG1), and FER have been shown to remodel cell walls and also generate feedback effects on the wall in root hairs and pollen tubes (*4*, *5*). Therefore, it will be interesting to investigate whether a similar mechanism also functions in hook development and in mediating defects stemming from a reduction in RG-II dimerization. RG-II is a complex component of the cell wall and its dimerization is essential for growth as evidenced by the *mur1* mutant hook phenotype. Nevertheless, the growth defects in the *mur1* mutant have remained enigmatic. Our findings now highlight a previously unrecognized impact of reduced RG-II dimerization on plant morphogenesis, revealed by disruption of differential growth (in apical hook development) in the *mur1* mutant due to modulation of hormonal pathways.

## MATERIALS AND METHODS

### Plant materials

The *Arabidopsis thaliana* ecotype Columbia (Col-0) was used as a wild-type control in all experiments. The following mutant lines were used in this study: *mur1-1* and *mur1-2* (*22*), *mur2-1* (*63*), *arf7arf19* (*64*), *the1-1* (*37*), *mur1 the1-1* (generated in this study), *bes1-D* (*34*), and *mur1 bes1-D* (generated in this study). The following fluorescent lines were used in this study*: DR5::Venus* (*65*), *mur1 DR5::Venus* (generated in this study), *35S::Lti6a-GFP* (*66*), *mur1 35S::Lti6a-GFP* (generated in this study), *proML1::ARF7-mCherry* (generated in this study), *mur1 proML1::ARF7-mCherry* (generated in this study), *PIN3-GFP* (*25*), *mur1 PIN3-GFP* (generated in this study), *PIN4-GFP* (*67*), *mur1 PIN4-GFP* (generated in this study) *AUX1-YFP* (*68*), *mur1 AUX1-YFP* (generated in this study), *LAX3-YFP* (*69*), *mur1 LAX3-YFP* (generated in this study), and *rlp44* (*40*) and *mur1rlp44* (generated in this study).

### Plant growth conditions

Plants were grown on ½ MS (Murashige and Skoog nutrient) medium (Duchefa) supplemented with 0.5% (w/v) sucrose, 0.8% plant agar (Duchefa), and 2.5 mM 2-morpholinoethanesulfonic acid (Sigma-Aldrich) buffered at pH 5.81 with KOH. After sterilization, *A. thaliana* seeds were stratified at 4°C for 2 days in darkness followed by 6 hours of white light exposure at 21°C. Seeds were germinated vertically in square petri dishes kept at 21°C for the desired amount of time. Boron supplementation was performed by adding boric acid (Sigma-Aldrich), and PBA (Sigma-Aldrich) was used to induce boron deficiency as described by (*23*) and (*24*). l-Fucose (Sigma-Aldrich), boric acid, and PBA were dissolved in Milli-Q water, filter sterilized, and finally added to ½ MS medium immediately before it was solidified.

### Kinematic analysis of apical hook development

Kinematic (time-lapse imaging) of apical hooks was performed on seedlings grown on vertical plates in darkness at 21°C illuminated with far infrared light (850 nm). Seedlings were photographed every 4 hours using a Canon D50 camera without the infrared light filter. Captured images were processed as described previously using ImageJ software (*70*).

### RNA isolation and quantitative real-time PCR analysis

Apical hooks were harvested from 2-day-old dark-grown seedlings and total RNA was extracted using the RNeasy Plant Mini kit (QIAGEN) according to the manufacturer’s protocol. Extracted RNA was subsequently treated with ribonuclease-free TURBO deoxyribonuclease (DNase; Life Technologies, Ambion) to remove DNA contamination. A 1-μg sample of DNase-treated RNA was then used to synthesize cDNA using an iScript cDNA Kit (Biorad). Quantitative real-time polymerase chain reaction (qRT-PCR) analyses were performed with a Roche Light Cycler 480 thermal cycler, and relative expression values were calculated using the Δ-*C*_t_ method. UBQ10 was used as a reference gene for all experiments mentioned in the manuscript. The sequences of primer pairs used in this work are listed in table S1.

### Genotyping of mutants

Genotyping of *Arabidopsis* mutant plants was performed using the goTaq master mix (Promega Biotech). The *arf7arf19* mutant (*64*), *the1-1* (*37*), *rlp44* (*40*), and *bes1-D* (*34*) genotyped as described previously and the *mur1-2* were genotyped using the primers listed in table S2.

### Confocal laser scanning microscopy

Samples were imaged using a Carl Zeiss LSM 780 confocal laser scanning microscope. Green fluorescent protein (GFP) and Venus were excited by 488- and 514-nm lasers, respectively, and respective fluorescence emissions were detected at 492- to 540-nm and 518- to 560-nm windows.

Identical confocal acquisition parameters (laser power, photomultiplier gain, offset, zoom factor, and resolution) were used for the wild type and *mur1-2*. The fluorescence intensity was quantified using ImageJ. For each experiment, *n* ≥ 10 seedlings and 60 to 70 cells in total were quantified.

### Time-lapse imaging for analysis of cell elongation

Apical hooks of dark-grown wild-type (±boron) and *mur1-2* (±boron) seedlings expressing the plasma membrane marker Lti6a-GFP were imaged on vertical plates using a Nikon AZ-C2 vertical macroconfocal microscope with a 5×/0.5 WD 15-mm macro-objective every 2 hours over an 8-hour period. Cell lengths were measured using ImageJ software. Changes in the lengths of individual cells between time points were used to calculate hourly growth rates. To compare cell elongation rates between the outer and inner sides of the apical hook, approximately 60 to 70 epidermal cells were analyzed from each side in three to five different seedlings per genotype.

### Analysis of RG-II boron bridging status in Arabidopsis hypocotyls

Hypocotyls of Arabidopsis seedlings 72 hours after germination were homogenized in 75% ethanol, stirred for 4 to 6 h at 20°C, washed three times in 96% acetone, and then twice in 100% acetone, and dried. An aliquot of the alcohol-insoluble residue (AIR) was treated with 1 M Na_2_CO_3_ at 4°C for 16 hours (which removes methylester groups), neutralized with acetic acid, washed in water followed by acetone, and dried. A sample (5.0 mg dry weight) of the de-esterified AIR was then digested in 1 ml of an *Aspergillus aculeatus* endo-polygalacturonase [Megazyme, http://www.megazyme.com/; previously dialyzed against pyridine/acetic acid/water (1:1:98, v/v); 5 U/ml] solution for 16 hours at 20°C. A portion (5 to 30 μl) of each digest was analyzed by polyacrylamide gel electrophoresis and the RG-II bands were silver stained (*71*) and scanned. RG-II dimer and monomer band luminosity were quantified by analyzing greyscale Photoshop files (psd), as described (*72*). Duplicate marker mixtures containing the RG-II dimer and monomer [1:1, w/w; purified from an *Arabidopsis* cell suspension culture (*72*)] were run on each gel and the corrected mean luminosity of their bands after scanning was used to calibrate the dimer:monomer (w/w) ratio of the experimental samples.

### Immunoblot analysis

For protein extraction, ca.75 hypocotyls of 4-day-old Col-0 and *mur1-2* seedlings with or without boron treatment were collected. Plant materials were frozen in liquid nitrogen, ground in a Retsch MM400 mill, and homogenized in 100 μl of ice-cold homogenization buffer consisting of 1% (v/v) SDS, 25 mM tris/HCl (pH 7.5), 150 mM NaCl, 10 mM dithiothreitol, and Roche Complete protease inhibitor (one tablet, 10 ml^−1^). The homogenate was then placed on ice for 30 min before being centrifuged twice (10 min, 16,000*g*) at 4°C. After adding 4× lithium dodecyl sulfate and sample reducing agent (10×), the samples were heated for 10 min at 70°C, centrifuged again, separated on 4 to 15% (v/v) SDS–polyacrylamide gel electrophoresis stain-free protein gel (Bio-Rad Laboratories), and blotted on Trans-Blot Turbo Mini PVDF Transfer Packs. Membranes were blocked at 4°C with 5% (v/v) Difco Skim Milk. For immunodetection, an anti-BES1 antibody at 1:5000 was used as the primary antibody, and donkey antirabbit (Merck) at 1:10,000 was used as the secondary antibody. For tubulin detection, anti-tubulin (Abcam) at 1:5000 was used as the primary antibody, and sheep antimouse (Merck) at 1:10,000 as the secondary antibody. Proteins were detected by the ChemiDoc MP Imaging System (Bio-Rad Laboratories). The ratio of dephosphorylated BES1 to total BES1 proteins was quantified on the basis of signal intensity. Loading was adjusted to an equal level based on the amount of tubulin. Signal intensities were determined with Image Lab (Bio-Rad Laboratories). The BES1 immunoblot analyses were done using four biological replicates.

### Generation of *proML1:ARF7-mCherry* transgenic plants

The *proML1:ARF7-mCherry* construct was generated similarly to earlier published (*20*). The coding sequence of *ARF7* (*At5g20730*) with first intron and 5′ flanking AscI (GGCGCGCC) and 3′ flanking KpnI (GGTACC) was synthesized (GENEWIZ Europe, Leipzig, Germany). The custom synthesized gene AscI-ARF7^+1st intron^-KpnI (3829 bp) was cloned into a plasmid vector pUC-GW-Kan (2626 bp, GENEWIZ). The restriction-based cloning was performed to generate translation fusion of *ARF7^+1st intron^* and *mCherry* downstream of the Arabidopsis ML1 promoter sequence (*73*) in pDONR223 (Invitrogen Life Technologies). pML1: ARF7^+1st intron^-mCherry was subsequently cloned into pMDC99 destination vector by Gateway LR Clonase mix (11791-019, Life Technologies). The resultant plant expression vector was transformed into *Agrobacterium tumefaciens* GV3101, transforming *Arabidopsis* Col-0 via floral dip (*74*). Transformed plants were selected on ½ MS agar plates supplemented with hygromycin-B (25 mg/ml) (Duchefa). Three independent transformants were chosen for further analyses.
