# Supplementary material for: Reduced RG-II pectin dimerization disrupts differential growth by attenuating hormonal regulation
Source: Sci Adv. 2025 Feb 12;11(7):eads0760. doi: 10.1126/sciadv.ads0760 (PMC11817947; doi:10.1126/sciadv.ads0760)
Supplement: Supplementary file 1 — Figs. S1 to S12 Tables S1 and S2 [file sciadv.ads0760_sm.pdf]

Science Advances
AAAS

Supplementary Materials for

Reduced RG-II pectin dimerization disrupts differential growth by attenuating hormonal regulation

Pawan Kumar Jewaria *et al.*

Corresponding author: Rishikesh P. Bhalerao, rishi.bhalerao@slu.se

Sci. Adv. **11**, eads0760 (2025)
DOI: 10.1126/sciadv.ads0760

This PDF file includes:

Figs. S1 to S12
Tables S1 and S2

Supplementary Materials

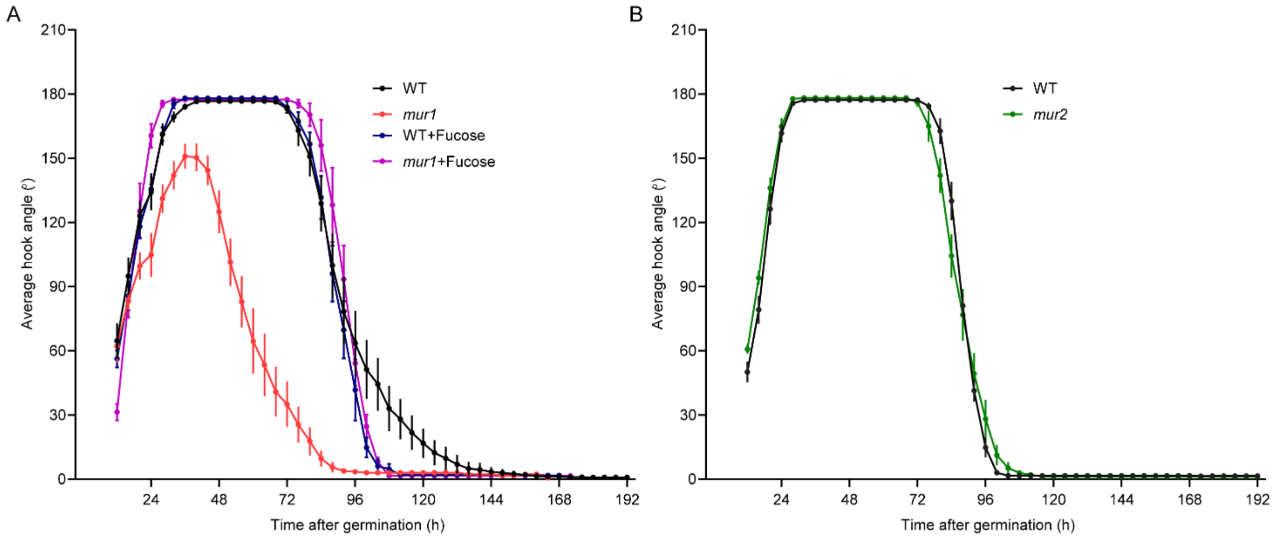

Figure S1. The *mur1* phenotype suppressed by exogenously added L-fucose and xyloglucan fucosylation mutant *mur2* displays normal apical hook development.
(A) Kinematics analyses of apical hook development in dark-grown wild-type (WT) and *mur1-2* seedlings without or after supplementation with 5 mM L-fucose. Error bars represent the SE of the mean (n ≥15).

(B) Kinematics analyses of apical hook development in dark-grown wild-type (WT) and *mur2* mutant seedlings. For each genotype, n ≥15. Error bars represent the SE of the mean.

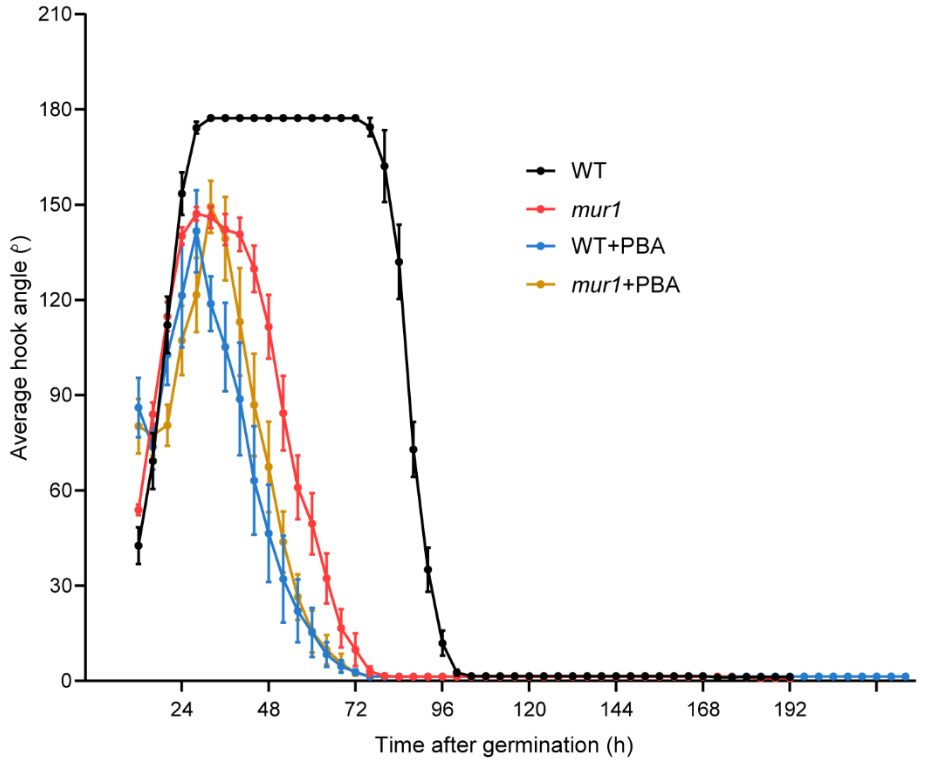

Figure S2. Treatment with the boronic acid derivative phenylboronic acid (PBA) causes severe hook defects in wild-type Arabidopsis, phenocopying the *mur1* mutant. Kinematics analyses of apical hook development in wild-type (WT) Arabidopsis seedlings grown in the presence of PBA (0.1 mM). For each genotype, n ≥15. Error bars represent the SE of the mean.

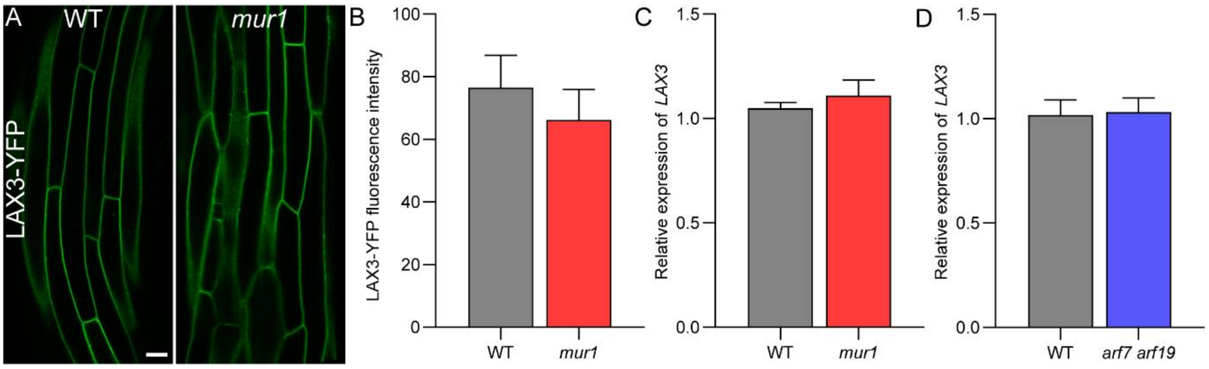

Figure S3. The *mur1* mutation does not affect *LAX3* transcript or PM levels. (A) Representative confocal images of plasma membrane-localized LAX3-YFP in the hypocotyls of WT and *mur1-2* mutant seedlings. The scale bar, 20 µm. (B) Quantification of plasma membrane fluorescence intensity of LAX3-YFP of epidermal cell in dark grown WT and *mur1-2* mutant. Quantification is an average of 5 cells each from 10 seedlings. Seedlings were imaged 48 h after germination. The plotted values are means ± SE (n ≥ 60-70 cells from 10 seedlings for each genotype. No significant differences were found according to Tukey's honest significant difference (HSD) test and Duncan's test. (C-D) The transcript level of the *LAX3* in the wild-type (WT) and *mur1-2* mutant (C) and WT and *arf7arf19* mutant seedlings (D). Ubiquitin was used as an internal control. Plotted values are averages for three independent biological replicates. No Significant difference was found according to Tukey's honest significant difference (HSD) test and Duncan's test.

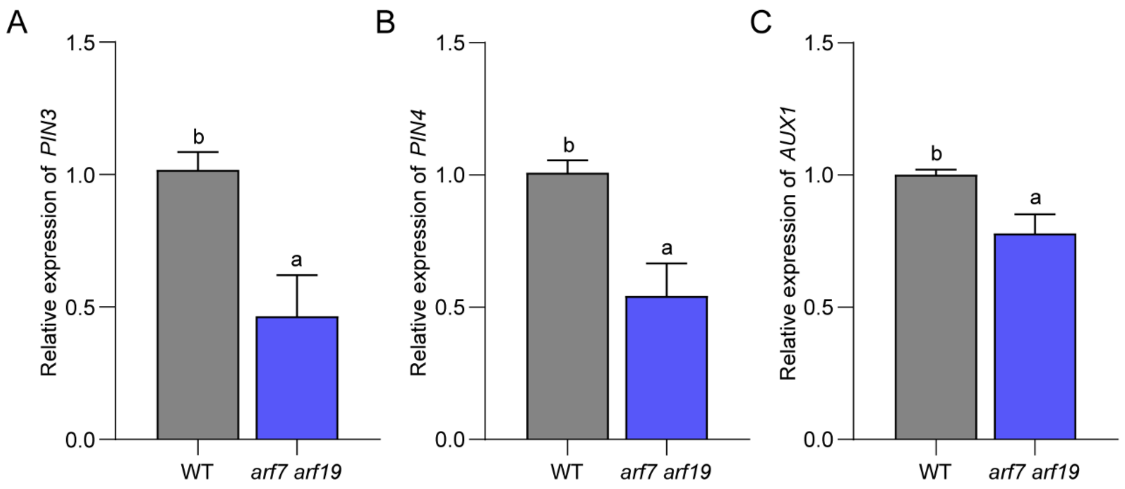

Figure S4. Expression of auxin carriers *PIN3*, *PIN4*, and *AUX1* are reduced in *arf7 arf19* mutant. The transcript levels of the auxin carriers (A) *PIN3*, (B) *PIN4*, and (C) *AUX1* in the wild-type (WT) and *arf7arf19* mutant seedlings. Ubiquitin was used as an internal control. Graphs represent averages of three biological replicates. Significant differences according to Tukey's honest significant difference (HSD) test and Duncan's test ($p < 0.05$) are indicated by different lowercase letters.

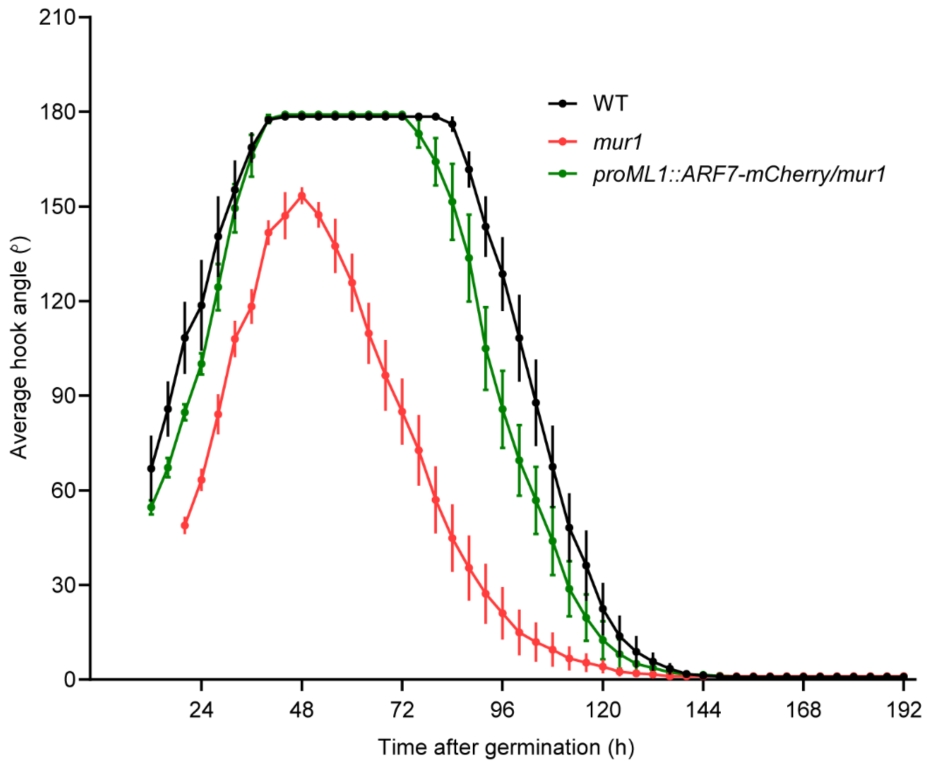

Figure S5. The Epidermal specific ML1 promoter driven by *ARF7-mCherry* suppresses *mur1* hook defect. Kinematics analyses of apical hook development in dark-grown wild-type (WT) *mur1-2,* and *pro::ML1-ARF7-mCherry/mur1-2* seedlings. For each genotype and condition, n ≥15. Error bars represent the SE of the mean.

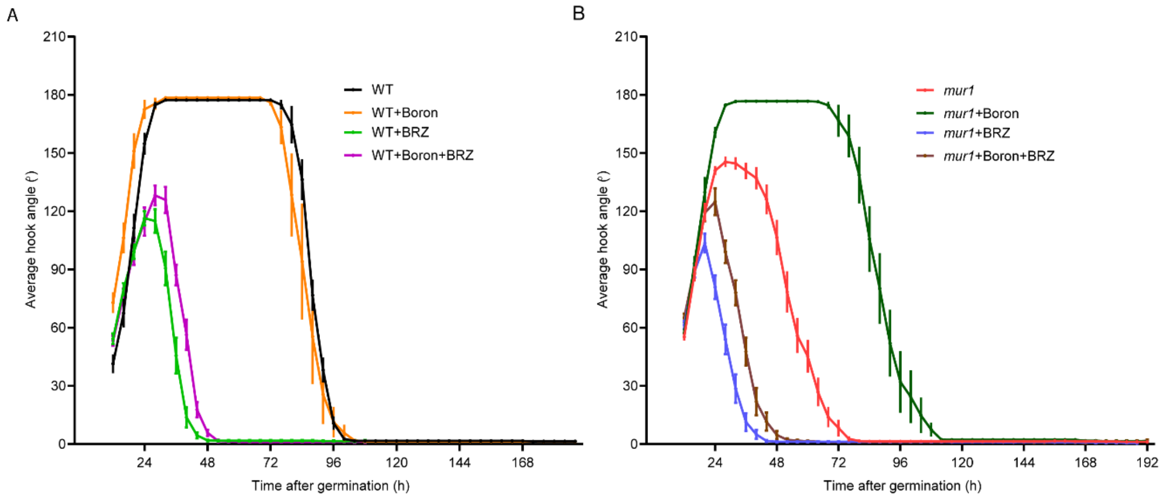

Figure S6. The BR biosynthesis inhibitor Brassinazole (BRZ) suppresses boron-mediated *mur1* mutant hook defect. Kinematics analyses of apical hook development in dark-grown wild-type (WT) and *mur1-2* seedlings in the presence or absence of the BR biosynthesis inhibitor BRZ (1 µM) without or with exogenously added boron (as 250 µM boric acid). For each genotype and condition, n ≥15. Error bars represent the SE of the mean.

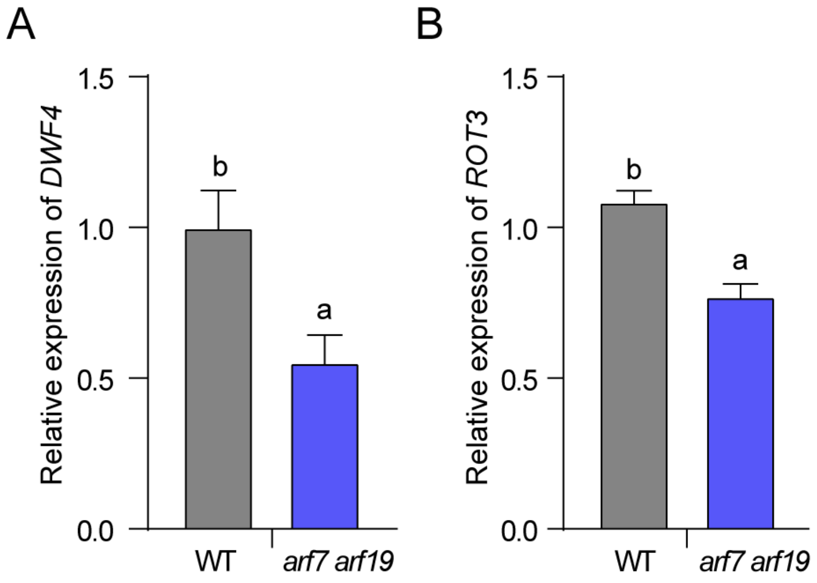

Figure S7. Attenuation of the brassinosteroid pathway downstream of ARF7/ARF19 leads to hook defects in the *mur1* mutant. (A-B) qRT-PCR analysis of *DWF4* and *ROT3* expression in wild-type (WT) and *arf7arf19* mutant seedlings. Ubiquitin was used as an internal control. Data represent averages of three independent biological replicates. Significant differences according to Tukey's honest significant difference (HSD) test and Duncan's test ($p < 0.05$) are indicated by different lowercase letters.

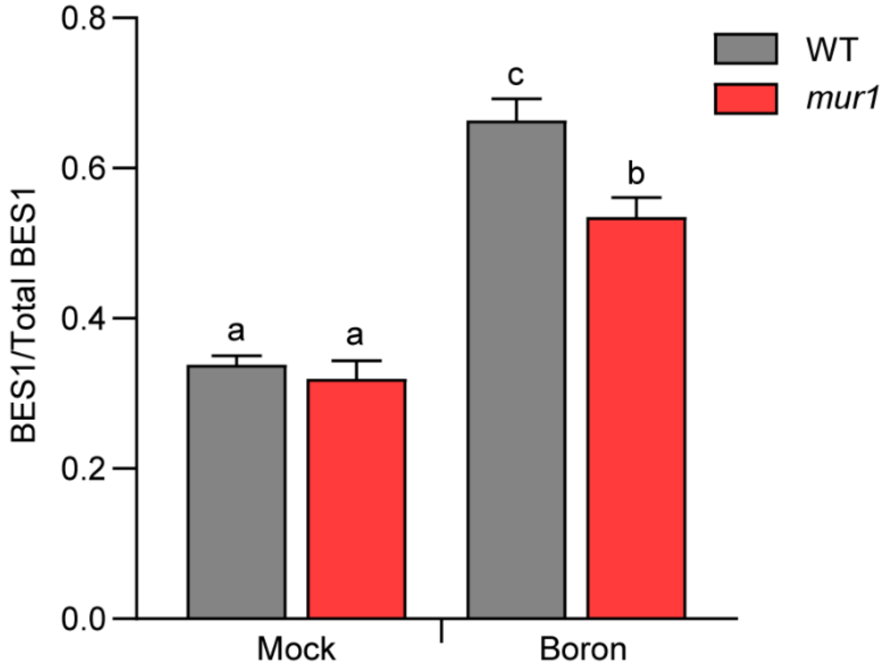

Figure S8. Boron enhances BR signaling. The ratio of dephosphorylated BES1 relative to total BES1 as a readout for BR signaling is plotted on the y-axis in wild-type (WT) and *mur1-2* mutant seedlings in the presence and absence of exogenously added boron.

Graphs represent averages of four biological replicates. Significant differences according to Tukey's honest significant difference (HSD) test and Duncan's test ($p < 0.05$) are indicated by different lowercase letters.

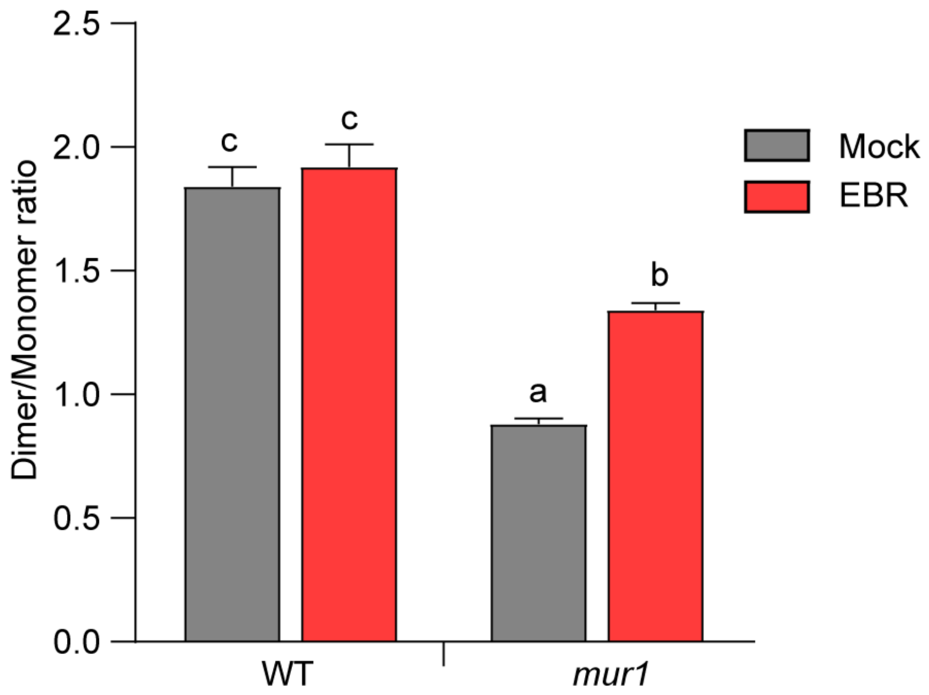

Figure S9. Brassinosteroid enhances RG-II dimerization in *mur1*. The addition of EBR (100 nM) increases dimerization of RG-II pectin in the *mur1* mutant. The y-axis denotes the ratio of RG-II dimer to monomer (mock-treated) or after EBR treatment (100 nM) in dark-grown wild-type (WT) and *mur1-2* hypocotyls.

The dimer-to-monomer ratio is the average of three biological replicates. Significant differences according to Tukey's honest significant difference (HSD) test and Duncan's test ($p < 0.05$) are indicated by different lowercase letters.

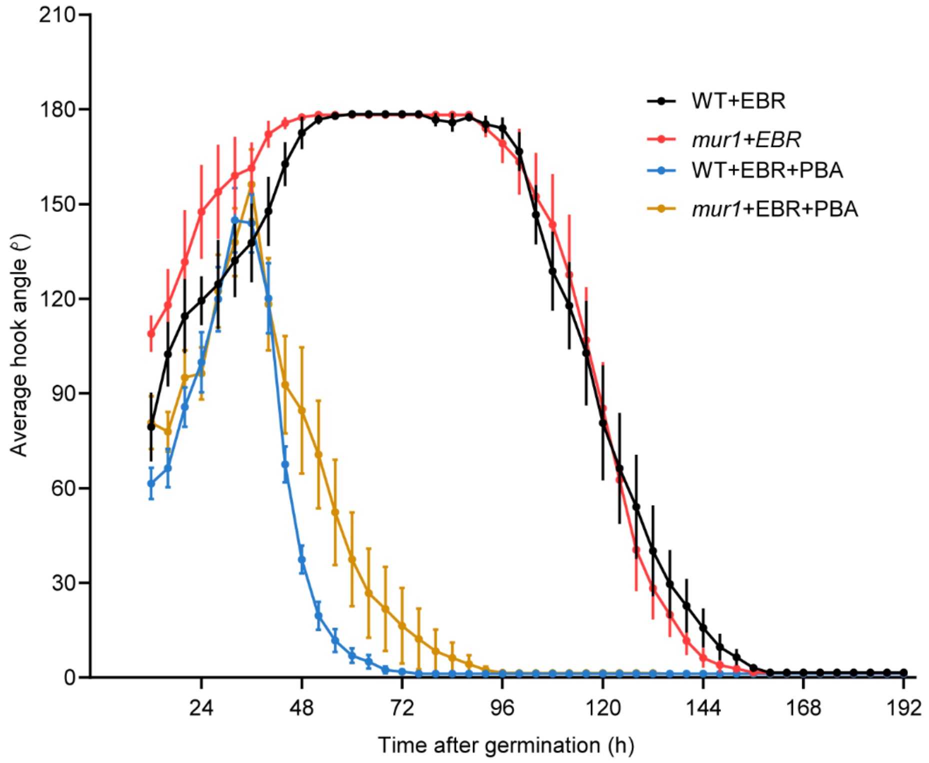

Figure S10. PBA blocks brassinosteroid-mediated suppression of *mur1*. Kinematics analysis of apical hook development in wild-type and *mur1-2* mutant seedlings after treatment with EBR (100 nM) and EBR with PBA (0.1 mM). For each genotype and condition, n ≥15. Error bars represent the SE of the mean.

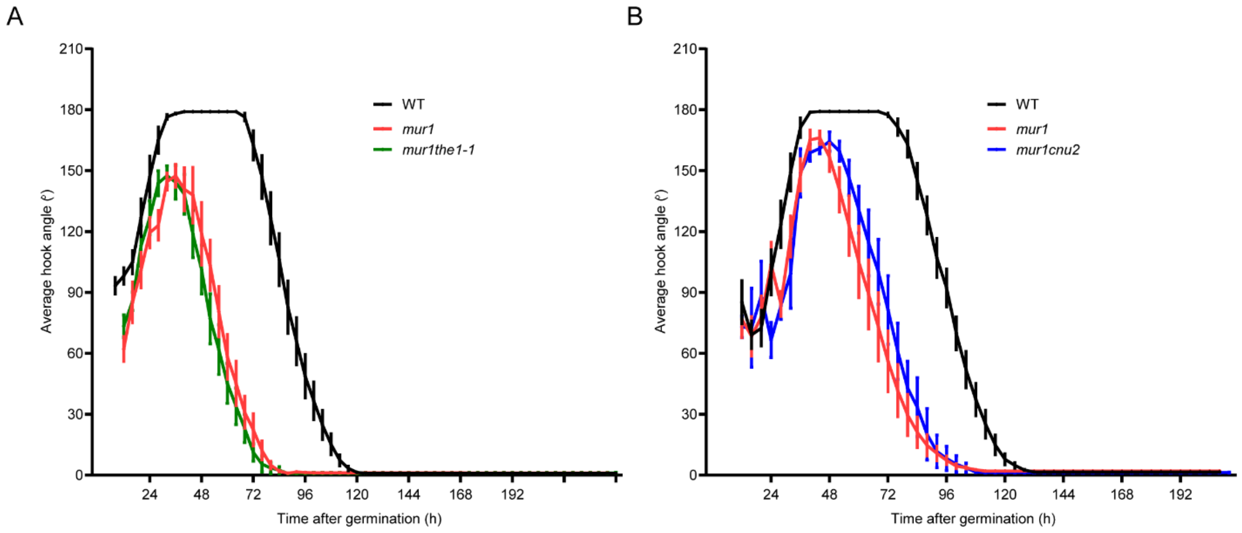

Figure S11. THESEUS1 or RLP44 independent transduction of cell wall defects in *mur1* mutant. Kinematics analyses of apical hook development in dark-grown wild-type (WT), *mur1-2*, *mur1-2the1-1*and *mur1-2rlp44* mutant seedlings. The *mur1-2the1-1* (A) and *mur1-2rlp44* (B) hook phenotype are the same as the *mur1-2* mutant phenotype. For each genotype, n ≥15. Error bars represent the SE of the mean.

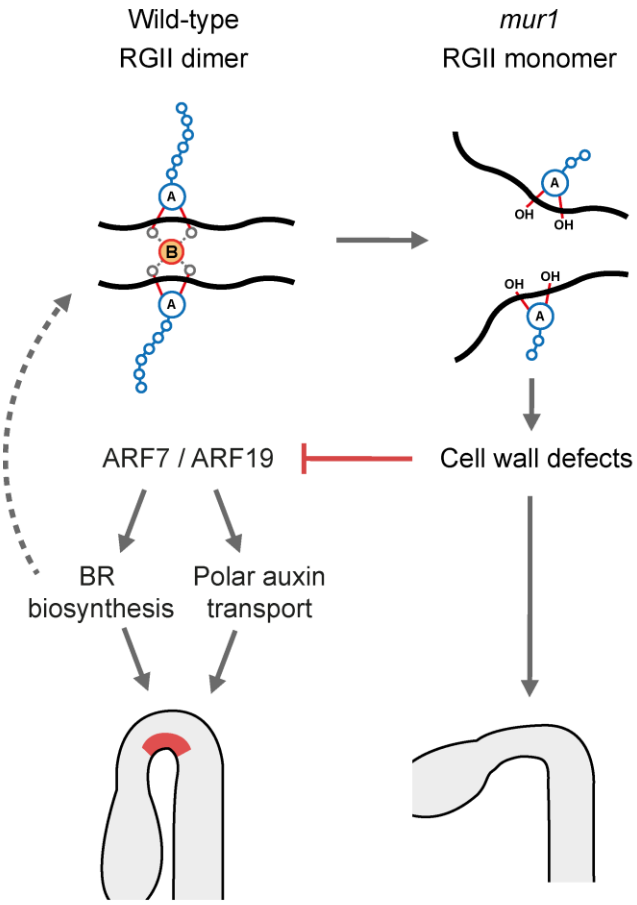

Figure S12. A schematic model for apical hook defects arising from reduced RG-II dimerization in the *mur1* mutant. In the wild-type apical hook, a high auxin response (depicted in red on the inner side of the hook) essential for asymmetric growth is generated by the activity of polar auxin transport components of the AUX/PIN family. The redundantly acting auxin response factors ARF7/ARF19 play a key role in hook development by regulating the expression of *AUX/PIN* as well as that of BR biosynthesis-related genes. Additionally, feedback from BR to the cell wall is indicated as a dashed arrow. In the *mur1* mutant, cell wall defects arising from reduced RGII dimerization, perceived independently of receptor-like kinase *THE1* or *RLP44*, result in the downregulation of *ARF7/ARF19* expression (shown as block arrow). Downregulation of *ARF7/ARF19* expression results in attenuation of polar auxin transport as well as expression BR biosynthesis-related genes perturbing the auxin response gradient, leading to disruption of asymmetric growth and hook defects.

Table S1: Sequence of primers used for expression analysis in qRT-PCR

**Oligo Name**	**Sequence (5'-3')**
ARF7-F	GCTCATATGCATGCTCCACA
ARF7-R	GCAATGCATCTCTGTCATATTTG
ARF19-F	CACCGATCACGAAAACGATA
ARF19-R	TGTTCTGCACGCAGTTCAC
MUR1-F	ATGGCGTCAGAGAACAACGGAT
MUR1-R	CGATGATCGACGGATCAGACCATG
DW4-F	CAGAGGATGAAGCAGAGATGAGTAAGAG
DW4-R	TCAAGATGCTCTTCCCTAAGCTCTTC
ROT3-F	ATTGGCGCGTTCCTCAGAT
ROT3-R	CAAGACGCCAAAGTGAGAACAA
PIN3-F	GCGTCAATAAAAACCCGAAA
PIN3-R	GGCGGTCTTTTGGTCTCTCTG
PIN4-F	TTGTCTCTGATCAACCTCGAAA
PIN4-R	ATCAAGACCGCCGATATCAT
AUX1-F	CAGGAATAGTACTTCAGATC
AUX1-R	GAACCAAGTAATCCATCAAG
LAX3-F	GGTAGGAGGATGGATGGGGA
LAX3-R	AAGTGTCGATCTGGCGAACA
UB10-F	ATCACCCTTGAAGTGGA
UB10-R	GAAACCACCACGAAGAC

Table S2: List of primers used for genotyping

**Oligo Name**	**Sequence (5'-3')**
arf7-LP	CAGCTAGATCGTTCGAAATGG
arf7-RP	AGCACATCACCATTTAGGTGC
arf19-LP	TTGCATTTGGAGTTGCTGAG
arf19-RP	CAAGGGCAAGTCCTTCAGAG
MUR1-2dCAPS F with EcoRII	GACAGCGACGTGAGATTGAGCAGCTAGGTTATAAACTT CGC
MUR1-2R	CGGATCATACCTGACGGAGTTCCTT
the1-1 F	AGCTTTTGGGTTTTCTTCGTTTTCC
the1-1 R	CTGTTTTGGAAAGTTATGTTTTGTGACTAG-3'
rlp44 F	AATCTACAAACTCTCACTCAC
rlp44 R	CTGACCGGATAATTCGTTATC
LB1	GCGTGGACCGCTTGCTGCAACT
LB3	TAGCATCTGAATTTCATAACCAATCTCGAT
